# α-Ketoglutarate Upregulates Collecting Duct (Pro)renin Receptor Expression, Tubular Angiotensin II Formation, and Na^+^ Reabsorption During High Glucose Conditions

**DOI:** 10.3389/fcvm.2021.644797

**Published:** 2021-06-04

**Authors:** Aarón Guerrero, Bruna Visniauskas, Pilar Cárdenas, Stefanny M. Figueroa, Jorge Vivanco, Nicolas Salinas-Parra, Patricio Araos, Quynh My Nguyen, Modar Kassan, Cristián A. Amador, Minolfa C. Prieto, Alexis A. Gonzalez

**Affiliations:** ^1^Instituto de Química, Pontificia Universidad Católica de Valparaíso, Valparaíso, Chile; ^2^Department of Physiology, School of Medicine, Tulane University, New Orleans, LA, United States; ^3^Laboratory of Renal Physiopathology, Institute of Biomedical Sciences, Universidad Autónoma de Chile, Santiago, Chile; ^4^Skaggs School of Pharmacy and Pharmaceutical Sciences, University of California, San Diego, San Diego, CA, United States; ^5^Department of Physiology, College of Medicine, University of Tennessee Health Science Center, Memphis, TN, United States

**Keywords:** prorenin receptor, diabetes, angiotensin, collecting duct, Kreb's cycle

## Abstract

Diabetes mellitus (DM) causes high glucose (HG) levels in the plasma and urine. The (pro)renin receptor (PRR) is a key regulator of renal Na^+^ handling. PRR is expressed in intercalated (IC) cells of the collecting duct (CD) and binds renin to promote angiotensin (Ang) II formation, thereby contributing to Na^+^ reabsorption. In DM, the Kreb's cycle is in a state of suppression in most tissues. However, in the CD, expression of glucose transporters is augmented, boosting the Kreb's cycle and consequently causing α-ketoglutarate (αKG) accumulation. The αKG receptor 1 (OXGR1) is a Gq-coupled receptor expressed on the apical membrane of IC cells of the CD. We hypothesize that HG causes αKG secretion and activation of OXGR1, which increases PRR expression in CD cells. This effect then promotes intratubular AngII formation and Na^+^ reabsorption. To test this hypothesis, streptozotocin (STZ)-induced diabetic mice were treated with or without montelukast (ML), an OXGR1 antagonist, for 6 days. STZ mice had higher urinary αKG and PRR expression along with augmented urinary AngII levels and Na^+^ retention. Treatment with ML prevented all these effects. Similarly, primary cultured inner medullary CD cells treated with HG showed increased PRR expression, while OXGR1 antagonist prevented this effect. αKG increases PRR expression, while treatments with ML, PKC inhibition, or intracellular Ca^2+^ depletion impair this effect. *In silico* analysis suggested that αKG binds to mouse OXGR1. These results indicate that HG conditions promote increased levels of intratubular αKG and OXGR1-dependent PRR upregulation, which impact AngII formation and Na^+^ reabsorption.

## Introduction

A hallmark of diabetic disease is the activation of the intrarenal renin–angiotensin system (RAS) (1–3). This system contributes to the development of hypertension by increasing intratubular angiotensin II (AngII)-dependent activation of Na^+^ transporters and thereby stimulating tubular renal Na^+^ reabsorption (4, 5). In most of the cases, patients with diabetes mellitus (DM) develop hypertension, which further increases their risk of kidney disease (6). Despite the suppressed plasma renin activity (PRA) observed in these patients, treatment with RAS inhibitors slows the progression of hypertension (7). Patients with DM show high plasma prorenin instead of active renin (8–10); indeed, plasma prorenin does not correlate with plasma renin concentrations and might predict microvascular damage (11). Animal models of type I DM showed augmented expression of prorenin in the renal collecting duct (CD) (2) and upregulation of the (pro)renin receptor (PRR) in the kidney (12, 13).

PRR binding to renin or prorenin induces a fourfold increase in the catalytic renin efficiency to convert angiotensinogen (AGT) to AngI and fully activates the non-enzymatically active prorenin (14). This suggests that upregulation of PRR in the CD results in further intratubular AngII formation. Increased expression and secretion of AGT by the proximal tubule cells (15, 16), as well as prorenin (2) and CD PRR (12) in diabetic conditions, further support this idea. Rats fed a high fat diet and given a low dose of streptozotocin (STZ) developed high blood pressure (17). Endothelial dysfunction STZ mice has been reported having mild effect on blood pressure during early phase of diabetes (18).

Diabetic conditions cause suppression of the tricarboxylic acid cycle (or Kreb's cycle) because oxaloacetate, an important component in the cycle, is instead channeled toward gluconeogenesis in the liver. Accumulation of nicotinamide adenine dinucleotide (NADH) also decreases the activity of α-ketoglutarate (αKG) dehydrogenase, leading to αKG accumulation and release (19). STZ-induced type I diabetic rats show increased urinary levels of αKG, citrate, and succinate in urine (19). Interestingly, diabetic rats show increased expression of facilitative glucose transporter GLUT1 in the CD (20). Upregulation of GLUT1 under high glucose (HG) conditions causes accelerated glucose uptake, glycolysis, and Kreb's cycle leading to a higher metabolic rate and accumulation of intermediaries of the Kreb's cycle such as succinate and αKG (21).

The recently deorphanized receptor for α-ketoglutarate (OXGR1) (22, 23) has been shown to increase intracellular Ca^2+^ (24). In mice, OXGR1 is expressed predominantly in kidney CD cells in the apical side of type B and non-A–non-B intercalated cells where it co-localizes with PRR (25). We have shown that PRR is upregulated by the activation of the AngII type 1 receptor (AT1R), a Gq-coupled receptor (26). As such, it is likely that the increased levels of plasma and urinary α-ketoglutarate described under HG conditions might reach the CD at physiological concentrations high enough to activate OXGR1, therefore stimulating signaling pathways to induce the expression of PRR.

In this study, we propose that in the early phase of STZ-induced diabetic hyperglycemia, αKG is augmented in the urine, activating ORXG1 receptor and consequently leading to the upregulation of PRR. This ultimately contributes to intratubular generation of AngII impacting on Na^+^ handling.

## Materials and Methods

### Experimental Animals and Sample Collections

All methods were performed in accordance with the Tulane Institutional Animal Care and the Bioethical Committee of the Pontificia Universidad Católica de Valparaiso, under international guidelines and regulations for animal use. Twelve-week-old male C57BL/6 mice were placed under the following conditions: light–dark cycle (12 h), temperature of 21°C, humidity of 50%, adequate ventilation, noise free, and food and water *ad libitum*. Mice were divided randomly into four groups: control (saline injection, normoglycemic mice, *n* = 9), streptozotocin-induced diabetic mice (200 mg/kg, single i.p. injection, *n* = 9), mice treated with OXGR1 antagonist montelukast (ML) (27) (5 mg/kg/day, i.p. and 4 h previous to STZ, *n* = 9), and STZ + ML (*n* = 9). STZ was injected after a 6-h fasting. Six hours was determined to be appropriate because a more prolonged fast may be inappropriate in mice, as it induces metabolic stress and enhances insulin action (28). Mice were considered diabetic if three consecutive blood glucose readings exceeded 250 mg/dl. Blood glucose was directly (not diluted) measured using ONETOUCH Ultra glucometer (LifeScan, catalog no. ZJZ8158JT, Milpitas, CA; reported result ranged 20–600 mg/dl) and also compared with regular glucometer Prodigy^©^ (https://www.prodigymeter.com/) demonstrating no differences in plasma glucose measurements. Renal tissue samples were analyzed after 6 days. Plasma and 24-h urine samples were collected from metabolic cages on day 6. A saline challenge was performed on day 5 to evaluate the effect of STZ or STZ plus OXGR1 antagonism on Na^+^ balance. Mice were injected i.p. with a volume of warmed isotonic saline equivalent to 10% of their body weight and placed immediately afterward in metabolic cages for urine collection. Results are expressed as the percentage of the injected sodium excreted over 5 h. On day 6, the animals were euthanized, and blood and kidneys were harvested. Urine samples from nine animals were collected into tubes containing an inhibitor cocktail [5 mmol/L of ethylenediaminetetraacetic acid (EDTA), 20 μmol/L of pepstatin A, 10 μmol/L of phenylmethylsulfonyl fluoride (PMSF), 20 μmol/L of enalaprilat, and 1.25 mmol/L of 1,10-phenanthroline]. After centrifugation at 4°C for 10 min at 1,000 *g*, urine was separated and applied to phenyl-bonded solid-phase extraction columns that had been prewashed with methanol followed by water. After sample application, angiotensin peptides were eluted from the solid-phase extraction column with 90% methanol. The eluates were collected, evaporated to dryness under vacuum, and stored at −20°C until radioimmunoassay was performed (29, 30). Angiotensin II was measured by using EIA Kit, Cayman, catalog no. A05880. The results are expressed in fmol/h. The α KG in urine samples and cell culture media was measured by Abcam α-ketoglutarate Assay Kit ab83431 (Abcam, Cambridge, UK). Creatinine was measured using Creatinine Analyzer 2 (Beckman Coulter, Inc., Fullerton, CA). Calphostin C and thapsigargin (Sigma-Aldrich) were used at 10 and 1 nM, respectively.

### Systolic Blood Pressure Measurements

Animals were trained daily for 4 days to become accustomed to the tail-cuff procedure using photoplethysmography. Eight to 12 consecutive pulse readings were recorded for each mouse in each measurement at day 0, 3, and 6 of treatment. All data were recorded using BP-2000 series II Blood Pressure Analysis System (Visitech System Inc.) before daily Montelukast injection as previously described above.

### Primary Cultures of Mouse IMCD Cells

In a different group of mice, after kidney excision, inner medullary tissues were digested in 10 ml of Dulbecco's modified Eagle's medium (DMEM)–Ham F-12, 20 mg of collagenase B, 7 mg of hyaluronidase, 80 mmol/L of urea, and 130 mmol/L of NaCl and incubated at 37°C under continuous agitation for 90 min. After centrifugation, the pellet was washed in prewarmed culture medium without enzymes [DMEM–Ham F-12, 80 mmol/L of urea, 130 mmol/L of NaCl, 10 mmol/L of HEPES, 2 mmol/L of L-glutamine, penicillin-streptomycin (10,000 U/ml), 50 nmol/L of hydrocortisone, 5 pM 3,3,5-triiodothyronine, 1 nmol/L of sodium selenate, and 5 mg/L of transferrin, without fetal bovine serum (FBS) (pH 7.4; 640 mosmol/kg of H_2_O)]. The resulting inner medullary collecting duct (IMCD) cell suspension was seeded in 3-mm Petri dishes. IMCD were divided and treated with normal glucose (5 mM D-glucose, NG), HG (25 mM), and mannitol (25 mM) during 24 h. No effects on PRR or GLUT1 expression levels were observed in mannitol-treated group (data not shown). We continued to explore the effect of NG or HG in the following experiments. Two hours before changing to HG conditions, the IMCD cells were pretreated with and without ML (10^−7^ M; Sigma Chemical Co.), which was dissolved in dimethyl sulfoxide (DMSO) and diluted to the final concentrations with phosphate-buffered saline (31). Experiments of αKG measurements in supernatant were assessed after 24 h incubation in NG or HG and measured by Abcam α-ketoglutarate Assay Kit ab83431 (Abcam, Cambridge, UK). A single dose of 0.2 mM of αKG was used for the acute experiments in IMCD cells (32).

### PRR and GLUT1 Transcripts Quantitation by Real-Time qRT-PCR

Total messenger RNA (mRNA) was isolated from mouse renal medullas or IMCD cells using RNeasy Mini Kit (Qiagen, Valencia, CA) according to the manufacturer's protocol. Total RNA was quantified using NanoDrop system. Quantitative real-time RT-PCR (qRT-PCR) was performed using the following primers: GLUT1, 5′-CAG CTG TCG GGT ATC AAT GC-3′, 3′-TCC AGC TCG CTC TAC AAC AA-5′; PRR, 5′-CAC AAG GGA TGT GTC GAA TG-3′, 3′-TTT GGA TGA ACT TGG GAA GC-5′; β-actin, 5′-ATC ATG AAG TGT GAC GTT GA-3′, 3′-GAT CTT CAT GGT GCT AGG AGC-5′. Results were presented as the fold change ratio between the levels of mRNA of the interest gene against β-actin (“housekeeping” gene) compared to control group (*n* = 6). Primers were obtained from IDT Company (https://www.idtdna.com).

### Immunoblotting Analyses

Forty micrograms of protein samples was electrophoretically separated on a precast NuPAGE 10% Bis-Tris gel (Novex) at 200 V for 45 min followed by semi-dry transference to a nitrocellulose membrane (Invitrogen) using iBlot (Invitrogen, Carlsbad, CA, USA). Blots were blocked at room temperature (RT) for 3 h, incubated overnight with specific primary antibody at 4°C, subsequently incubated with the corresponding secondary antibodies (1:5,000 dilutions), at RT for 45 min, and then analyzed by normalization against β-actin, which was used as a housekeeping gene. PRR protein levels were detected using a polyclonal rabbit anti-PRR (*ATP6AP2*, 1:200; Cat. no. HPA003156, Sigma-Aldrich) that recognizes the intracellular segment and the ectodomain (33). GLUT1 was detected using rabbit anti-GLUT1 (1:200; Cat. no. SAB4200519, Sigma-Aldrich). For characterization of IMCD, we used anti- aquaporin (AQP)-2 antibody at 1:400 dilutions (Cat. no. 178612 Calbiochem, San Diego, CA) and NKCC cotransporter 1:500 dilutions (Cat. no. 51791, Abcam, UK). Immunoblots are presented in each figure as representative images. Results are presented as the ratio of PRR or GLUT1 vs. β-actin as fold change of control. Tissue analysis was performed by using six animals per group and three to five independent experiments for Western blot analysis.

### Immunofluorescence in Kidney Tissues and Primary Cultures of IMCD Cells

Three-micrometer kidney slides were fixed and stained with anti-PRR at 1:200 dilutions (ATP6AP2, Cat. no. HPA003156, Sigma-Aldrich, MO, USA), anti-GLUT1 1:100 dilutions (Cat. no. SAB4200519, Sigma-Aldrich), and anti-OXGR-1 1:100 dilutions (Cat. no. PA5-67872, Invitrogen, CA). For cultured cell immunofluorescence, 50–60% subconfluent IMCD cells cultured in chamber slides (Nalge Nunc) were fixed in cold methanol for 20 min, blocked with PBS-Tween (0.1%) plus bovine serum albumin (BSA) (3%) for 1 h, stained with the following antibodies: anti-aquaporin-2, AQP-2 (Cat. no. 178612, Calbiochem, San Diego, CA), anti-PRR (ATP6AP2, 1:100 dilutions; Cat. no. HPA003156, Sigma-Aldrich, MO, USA), anti-anion exchanger type 1, AE1 (Cat. no. AE11-A, Alpha Diagnostic Intl, San Antonio, TX), and anti-OXGR-1, 1:100 dilutions (Cat. no. PA5-67872, Invitrogen, CA) and detected with Alexa Fluor 488 or 594 conjugated to antirabbit immunoglobulin G (IgG) (Invitrogen, Life Science, Co.). The slides were mounted with ProLong® Gold with 4,6-diamidino-2-phenylindole dihydrochloride (DAPI) for nuclei staining. The images were obtained using a Nikon Eclipse-50i immunofluorescence microscope (Nikon Eclipse-50i, Japan) and were digitalized using the NIS-Elements BR version 4.0 from Nikon). Negative controls were obtained by omission of the specific primary antibody.

### *In vitro* Ca^2+^ Measurements

Cell suspensions (8 × 10^5^ cells/ml) were loaded with Fura-2 AM (5 μM) and incubated for 30 min at room temperature and protected from light and 37°C. Then, cells were washed with 1 × PBS and suspended. A volume of 500 μl was added in a quartz cell to measure fluorescence in Fluoromax-2 spectrofluorometer (Instruments SA, Edison, NJ). The [Ca^2+^] was calculated as: [Ca^2+^]i (nM) = Kd × [(R – R_min_)/R_max_ – R)] × Sfb, where Kd (for Ca^2+^ binding to Fura-2 at 37°C) = 225 nM, R = 340/380 ratio, R_max_ = 340/380 ratio under Ca^2+^-saturating conditions, R_min =_340/380 ratio under Ca^2+^-free conditions, and Sfb = ratio of baseline fluorescence (380 nm) under Ca^2+^-free and Ca^2+^-bound conditions.

### *In silico* Analysis of the Interaction of αKG and Mouse OXGR1

*In silico* analysis was performed to evaluate the interaction of αKG and mouse OXGR1 (GPR99) using the free-online servers I-TASSER, ProSA-Web, and MolProbidity (Duke University) for structure modeling, since there is no available structure for mouse GPR99. Docking model (αKG and mouse OXGR1 interaction) was performed using free-online software Swiss-Dock. Multiple sequence alignment of GPR99 and GPR91 was performed using software Clustal Omega (https://www.ebi.ac.uk/Tools/msa/clustalo).

### Statistical Analyses

Results are expressed as mean ± SEM. Grubb's test was used to detect outliers in univariate data assumed to have come from a normally distributed population. Comparisons between groups were performed using one-way ANOVA and Tukey's posttest. *p* ≤ 0.05 values were considered statistically significant.

## Results

### STZ-Induced Hyperglycemia Is Associated With Increased Urinary α-Ketoglutarate

**Figure 1:**
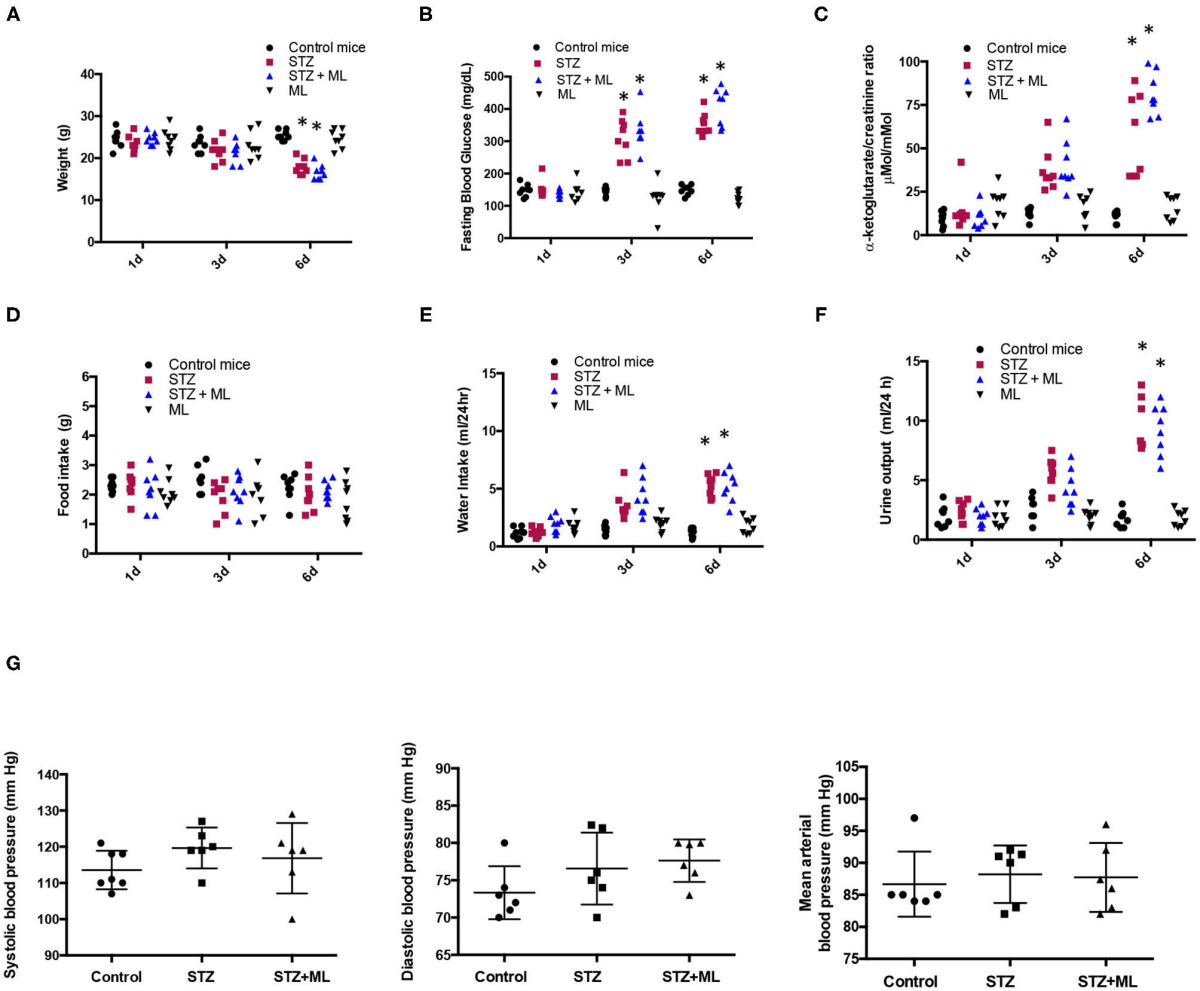
Physiological parameters in control mice and streptozotocin-induced diabetic mice with or without treatment with montelukast (ML), an OXGR1 antagonist. **(A)** Body weight was reduced after 6 days of streptozotocin (STZ) treatment. **(B)** Fasting glucose levels were augmented on days 3 and 6 in STZ and STZ + ML mice. **(C)** After 6 days, a significant increase in urinary levels of αKG (assessed by αKG/creatinine ratio) was detected in STZ and STZ + ML mice. **(D)** A slight but not significant reduction was observed in food intake. **(E)** Water intake was augmented in STZ and STZ + ML groups. **(F)** Urine output was also augmented in STZ and STZ + ML group. **(G)** Systolic, diastolic, and mean arterial blood pressure was not changed at the end of the treatment. *p < 0.05 vs. control group (*n* = 7–9).

As shown in Figure 1A, STZ mice have reduced body weight at day 6 (17.8 ± 0.8 vs. control mice, 23.3 ± 0.4 g, *p* < 0.05). Mice treated with the OXGR1 antagonist ML also showed a significant reduction in body weight (16.2 ± 0.6 g, *p* < 0.05 vs. control mice). Reduction in body weight was also confirmed and standardized by measuring body weight vs. tibia–length ratio. The data showed a less evident loss in body weight in STZ mice (STZ, 1.153 ± 0.017 vs. control, 1.333 ± 0.045, *p* < 0.05) and in STZ + ML group (STZ + ML, 1.193 ± 0.042 vs. control, 1.333 ± 0.045, *p* = 0.23). Mice treated only with ML did not show changes in body weight (24.5 ± 0.7 g, *p* = 0.23 vs. control mice). Fasting blood glucose levels were significantly higher on day 6 in STZ-induced hyperglycemic mice (354 ± 12 mg/dl, *p* < 0.05 vs. controls) and STZ + ML mice (410 ± 20 mg/dl, *p* < 0.01 vs. controls) compared to controls (149 ± 5 mg/dl). Fasting blood glucose was not altered in mice treated with ML when compared to normoglycemic control mice (Figure 1B). Urinary levels of αKG assessed by αKG/creatinine ratio were significantly augmented in STZ and STZ + ML groups on day 6 as compared to controls (Figure 1C). No differences in plasma creatinine (controls, 0.45 ± 0.11; STZ, 0.53 ± 0.13; STZ + ML, 0.47 ± 0.05 mg/dl, *p* = 0.40) or blood urea nitrogen (controls, 12.5 ± 2.8; STZ, 20.1 ± 4.6; STZ + ML, 19.1 ± 5.7 mg/dl, *p* = 0.33) were found among the groups. Urine protein/creatinine ratio was not different among groups (controls, 2.2 ± 0.3; STZ, 4.7 ± 3.3; STZ + ML, 2.2 ± 1.3 mg/dl, *p* = 0.21). No effects were observed in ML group.

Since STZ animals had reduced body weight, we evaluated food intake in 24 h metabolic cages measurements. No differences were found in food intake on days 1, 3, and 6 between controls, STZ, and STZ plus ML (Figure 1D). As shown in Figure 1E, water intake was significantly greater in diabetic mice (STZ alone and STZ + ML treatment) compared to control mice on day 6. No effect was observed in the ML group. Of note, diuresis was significantly higher in STZ mice and STZ + ML group (Figure 1F) showing a negative 24-h water intake vs. urine output balance at day 6. The treatment with ML alone did not affect urinary volume. Finally, a new set of experiments was performed to measure systolic and diastolic blood pressure. We observed a slight but not statistically significant (*p* = 0.081) increase in systolic blood pressure in STZ mice (Figure 1G).

### Increased mRNA and Protein Levels of PRR in Renal Medullary Tissues of STZ Mice Are Blunted by OXGR1 Antagonism

**Figure 2:**
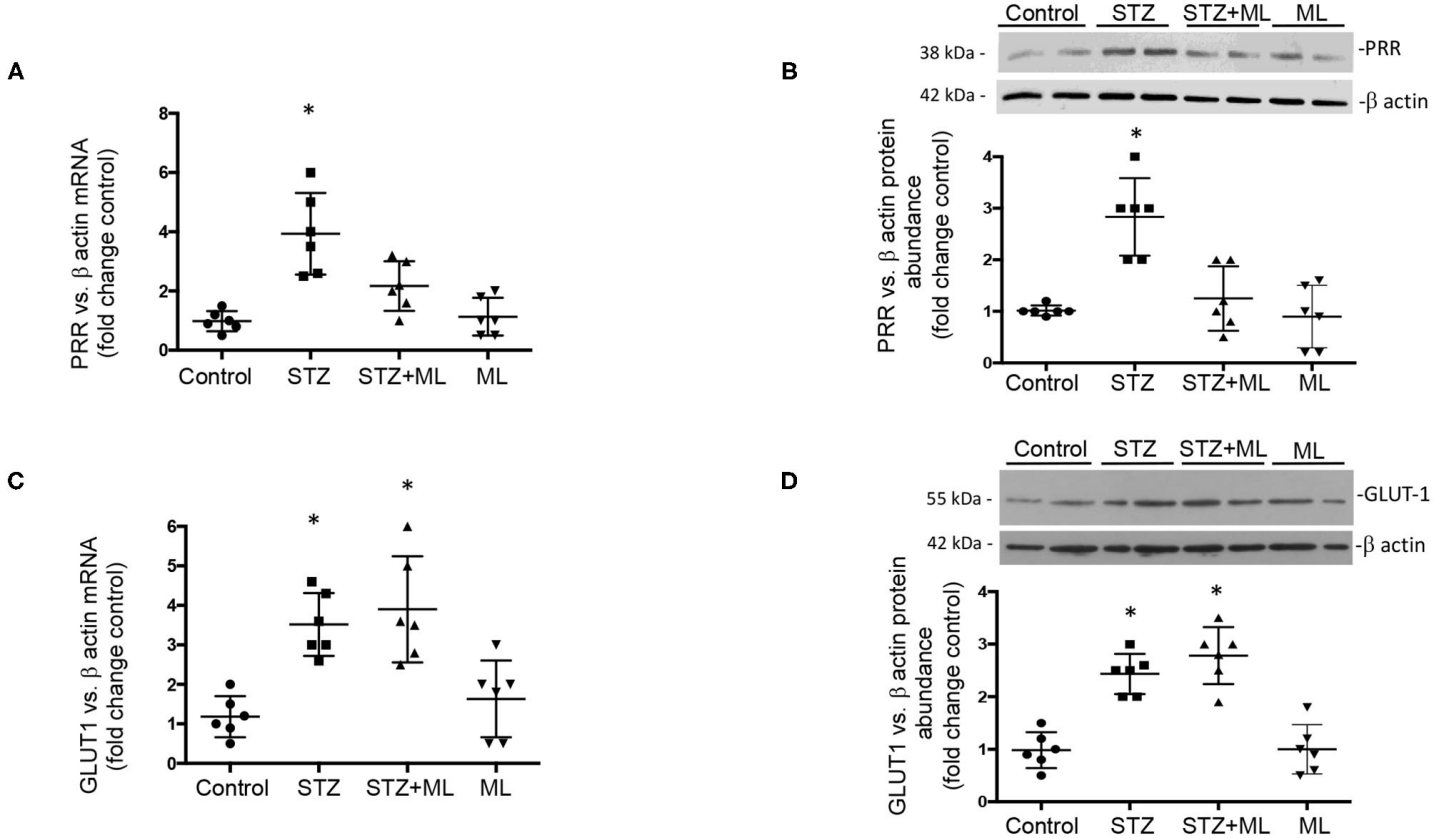
**(A)** After 6 days of a single streptozotocin (STZ) administration, (pro)renin receptor (PRR) messenger RNA (mRNA) levels were augmented in the renal medulla of STZ mice. **(B)** Same effect was observed in PRR protein abundance. Treatment with OXGR antagonist montelukast (ML) in STZ mice prevented the upregulation of PRR mRNA and protein. **(C)** GLUT1 mRNA and **(D)** protein levels were increased in the renal medulla of STZ mice, as well as in STZ and STZ + ML groups. ML alone did not show significant changes on PRR or GLUT expression. *p < 0.05 vs. control group (*n* = 6).

After 6 days of single STZ administration, PRR mRNA levels were augmented in STZ mice (3.7 ± 0.6 vs. control 1.0 ± 0.1, *p* < 0.05), while ML prevented the increase in PRR transcript levels (1.7 ± 0.4, *p* = 0.08 vs. controls, Figure 2A). The same effect was observed in PRR protein abundance (STZ, 3.8 ± 0.5 vs. controls, 1.0 ± 0.2, *p* < 0.05). Treatment with OXGR antagonist ML in STZ mice prevented the upregulation of PRR protein (1.7 ± 0.4, *p* = 0.32 vs. controls, Figure 2B). We next evaluated the expression of GLUT1 in STZ-induced HG conditions in the renal medulla and the effect of OXGR1 antagonist ML. Both treatments STZ and STZ + ML caused an increase in the mRNA (3.5 ± 0.5 vs. 1.1 ± 0.2, *p* < 0.05) and protein levels (2.4 ± 0.2 vs. 1.0 ± 0.1, *p* < 0.05) of GLUT1. ML alone did not show significant changes on GLUT1 abundance (Figures 2C,D).

### Augmentation of Urinary AngII and Decreased Na^+^ Excretion Was Attenuated by OXGR1 Antagonism in STZ Mice

**Figure 3:**
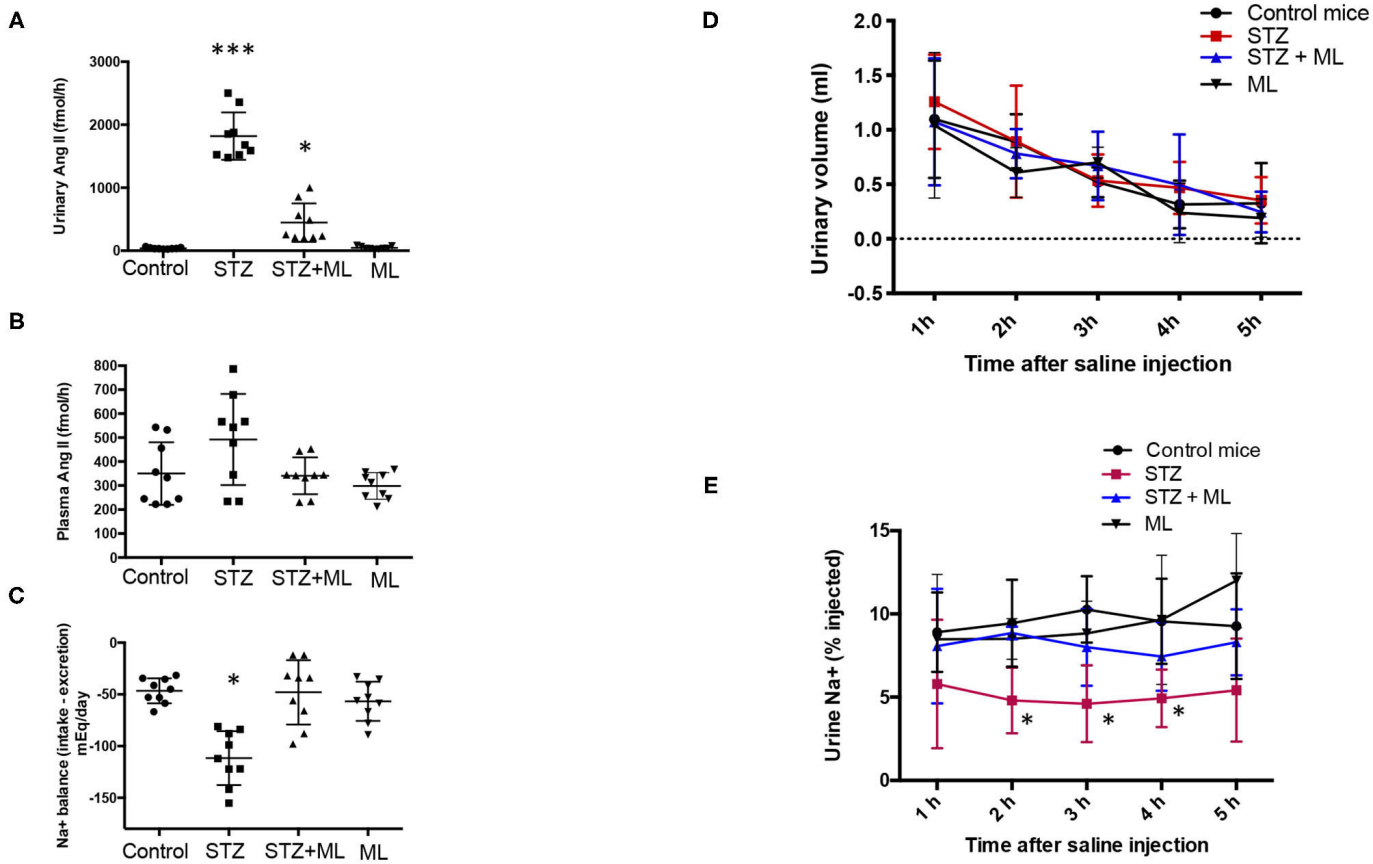
Streptozotocin (STZ) mice showed augmented urinary AngII excretion and Na^+^ retention that was prevented by OXGR1 pharmacological blockade. **(A)** AngII was significantly augmented in STZ mice (*p* < 0.001 vs. control group). Although mice treated with the OXGR1 antagonist montelukast (ML) also showed significant increases in AngII levels, this effect was less evident (*p* < 0.05). Treatment with ML alone in normoglycemic mice did not significantly change AngII excretion. **(B)** No changes were observed in plasma AngII. **(C)** Na^+^ balance assessed on day 6 demonstrated that STZ mice retained Na^+^. ML treatment in STZ mice blunted this effect. Saline challenge was performed to determine the percentage of injected Na^+^ that would be excreted. **(D)** Urine samples collected every hour showed a continuous decrease in total volume with no significant differences between controls and STZ or STZ + ML treated mice. **(E)** Na^+^ concentration of these samples was measured in order to calculate the percentage of Na^+^ excreted after saline (0.9% NaCl) injection. STZ mice showed a reduced percentage of injected Na^+^ in urine samples, demonstrating increased Na^+^ retention. This effect was less evident in STZ + ML group. Significant values were observed only at 2, 3, and 4 h as compared to controls. *p < 0.05 vs. control group, ***p < 0.001 vs. control group, *n* = 9.

Because downregulation of PRR expression in the collecting ducts may decrease intratubular AngII formation, we evaluated urinary AngII excretion. AngII levels were greatly augmented in STZ mice (STZ, 1890 ± 178 fmol/h vs. controls, 40 ± 7 fmol/h, *p* < 0.05). This augmentation effect was not seen in mice that received ML treatment (560 ± 130 fmol/h, *p* < 0.05 vs. STZ group). Treatment with ML alone in normoglycemic mice did not show significant changes in AngII excretion (Figure 3A). Because intratubular AngII formation may impact on intratubular Na^+^ handling, we evaluate the balance between Na^+^ intake and excretion on day 6. STZ causes Na^+^ retention, while ML prevented this effect (Figure 3B). We next performed a saline challenge to determine the percentage of injected Na^+^ that could be excreted by normoglycemic mice, STZ mice, and mice subjected to STZ plus ML treatment. As shown in Figure 3C, urine samples collected every hour showed a progressive decrease in total volume. Na^+^ concentration of these samples was measured to calculate the percentage of Na^+^ excreted after saline (0.9% NaCl) injection. As shown in Figure 3D, STZ mice had lower percentages of injected Na^+^ in their urine, suggesting increased Na^+^ retention. This effect was less evident in STZ + ML group. Significant values were observed only at 2 and 3 h as compared to controls.

### OXGR1 Co-localizes With PRR in Inner Medullary Collecting Duct Cells

**Figure 4:**
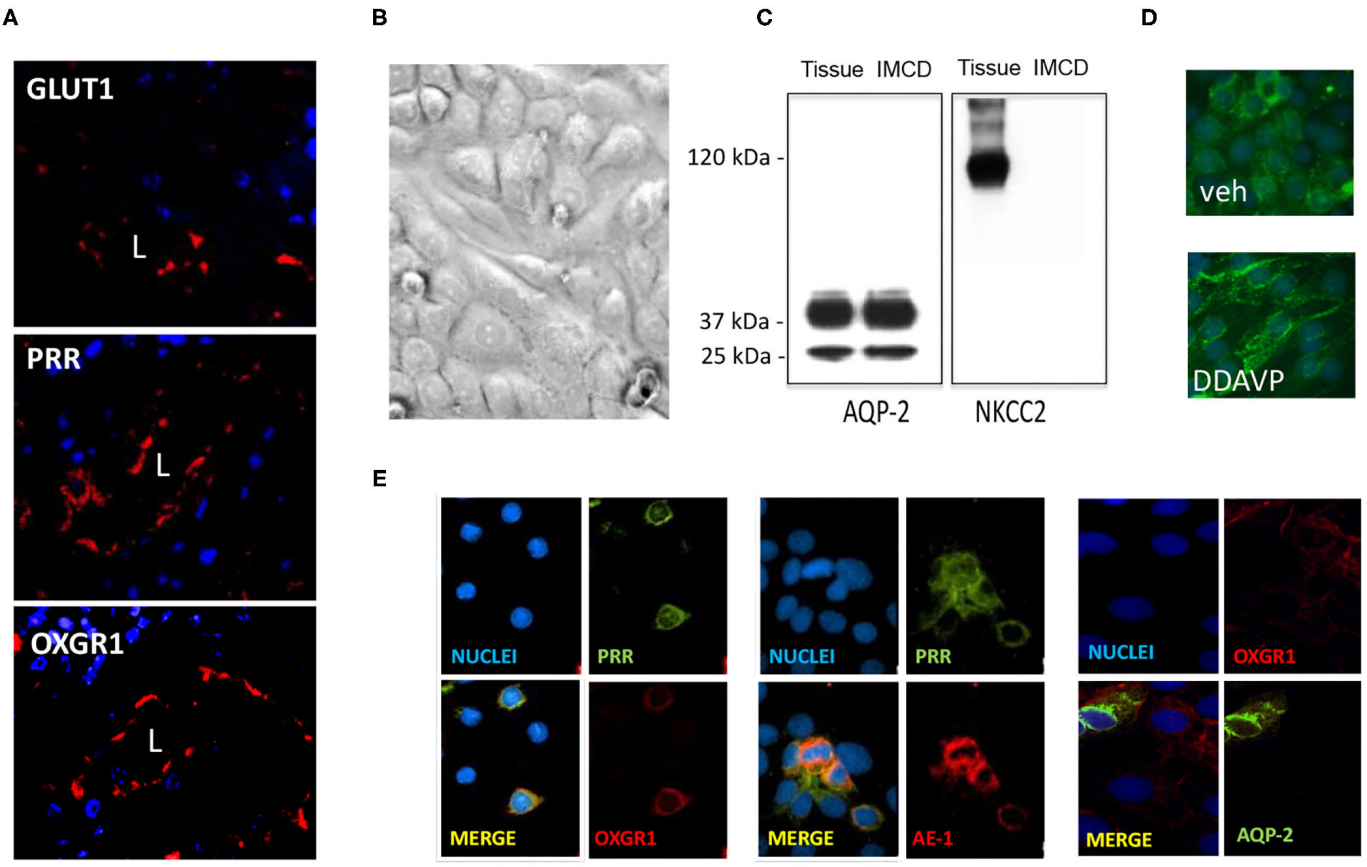
**(A)** Staining of whole kidney sections showed the presence of GLUT1, (pro)renin receptor (PRR), and OXGR1 (red staining). L indicates lumen. Nuclei are stained with 4,6-diamidino-2-phenylindole dihydrochloride (DAPI). **(B)** Inner medullary collecting duct (IMCD) cells were grown until reach confluence and assessed for the presence of NKCC to rule out the presence of cortical tissues cells. Western blot analysis showed the presence of aquaporin (AQP)-2 in homogenates of total kidney tissue and IMCD cells. **(C)** NKCC was absent in IMCD. Functional IMCD cells were assessed by the activation of the vasopressin V2 with a V2 receptor agonist (DDAVP, 10^−8^ M). **(D)** After 45 min, AQP-2 immunofluorescence was mostly detected in plasma membrane. Coimmunostaining demonstrated the coexistence of OXGR1 and PRR, which also co-localizes with AE-1, a marker of intercalated cell. **(E)** OXGR1 did not co-localizes with AQP-2, a marker of principal cell.

GLUT1, PRR, and OXGR1 were detected in inner medullary tissues (Figure 4A). IMCD cells were grown until confluence (Figure 4B) and assessed for the presence of sodium potassium 2-chloride transporter, NKCC, to rule out the presence of cortical tissues cells. Western blot analysis showed the presence of AQP-2 in homogenates of total kidney tissue and IMCD cells. NKCC was absent in IMCD (Figure 4C). To further evaluate the functional responses of IMCD cells to the activation of the vasopressin V2 receptor treatment, IMCD cells were treated with desmopressin (DDAVP, 10^−8^ M). After 45 min AQP-2 immunofluorescence was mostly detected in plasma membrane (Figure 4D). Coimmunostaining demonstrated the coexistence of PRR and OXGR1 and PRR, which also co-localizes with AE-1, a marker of intercalated cell. As observed in Figure 4E, 35–40% of the total cells in each microscopic correspond to intercalated cells as described by Kim et al. (34), which explains why PRR and OXGR1 are present only in few cells in the microscopic culture's fields. OXGR1 did not co-localizes with AQP-2, a marker of principal cell (Figure 4E).

### Treatment With αKG Increases Intracellular Ca^2+^ During Normal Glucose Conditions and HG Promotes αKG Accumulation in Cell Culture Media From Inner Medullary Collecting Duct Cells

**Figure 5:**
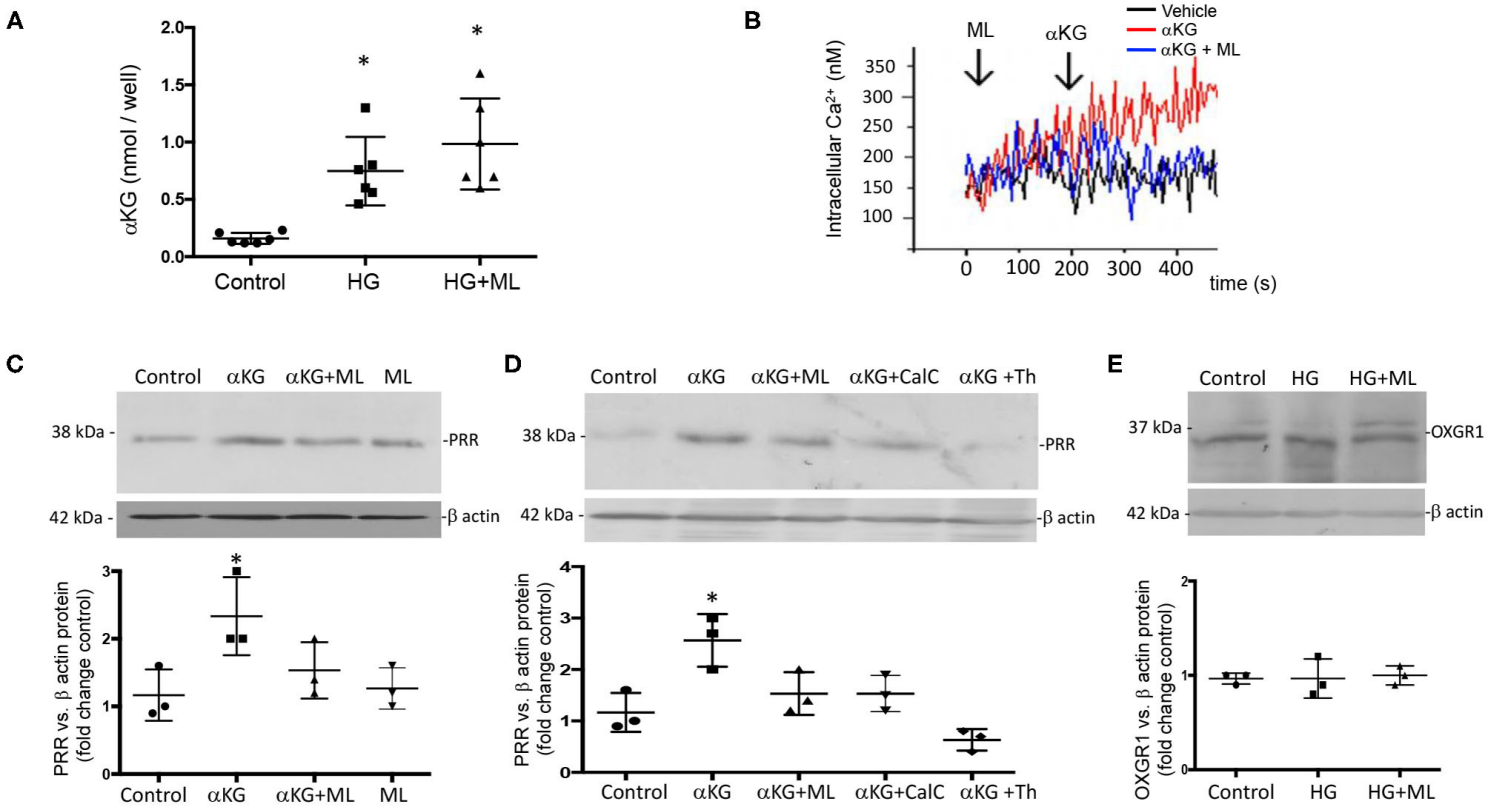
Evidence of the effect of αKG on (pro)renin receptor (PRR) expression and intracellular signaling pathways. **(A)** Incubations with HG during 24 h increased in αKG accumulation in inner medullary collecting duct (IMCD) cell culture media. HG + ML did not cause αKG accumulation (*p < 0.05 vs. control group, *n* = 5). **(B)** Functional assessment of cultured IMCD cells demonstrated that αKG induced a rise in intracellular Ca^2+^ that was blunted by OXGR1 antagonist montelukast (ML). This suggest that the mechanism involves the activation of OXGR1. **(C)** IMCD incubated with αKG 0.2 mM for 16 h showed increased levels of PRR; this effect was blunted by ML. **(D)** Increased PRR levels was suppressed by PKC inhibitor calphostin C (10 nM) or intracellular Ca^2+^ depletion using thapsigargin (1 μM). **(E)** OXGR1 expression did not change in HG conditions. For Panels **(C–E)**; *p < 0.05 vs. control group, *n* = 3.

Because OXGR1 is a Gq-coupled receptor that stimulates intracellular Ca^2+^ release, we evaluated the responses of cultures from IMCD cells to αKG treatment (10^−7^ M) on intracellular Ca^2+^ concentrations by using IMCD cells preloaded with Fura-2 AM (see *Methods* for details). As shown in Figure 5A, αKG treatment at 180 s causes an increase in intracellular Ca^2+^ concentrations from ~150 to ~300 nM at 400 s. IMCD cells previously treated with ML did not show increased intracellular Ca^2+^. No effects were seen in the presence of ML alone (data not shown). Then, we evaluated the effect of 24-h incubations under HG conditions on αKG levels in cell culture media. Figure 5B shows that αKG secretion is increased in the HG group (0.82 ± 0.35 nmol/well vs. control 0.22 ± 0.02 nmol/well, *p* < 0.05), as well as in HG + ML group (1.23 ± 0.42 nmol/well vs. control 0.22 ± 0.02 nmol/well, *p* < 0.05). ML alone has no effect on αKG levels. We next evaluated the effect of αKG treatment on PRR expression after 24 h. As shown in Figure 5C, αKG increased PRR protein abundance (2.6 ± 0.2 vs. 1.1 ± 0.2, *p* < 0.05), while pretreatment with ML blunted this effect (1.6 ± 0.2, *p* = 0.23 vs. control). OXGR1 activates PKC signaling pathways (35). We block the activity of PKC by using 10 nM of calphostin C, 30 min before αKG treatment. As shown in Figure 5D, Calphostin C prevented the increase in PRR (1.5 ± 0.2, *p* = 0.13 vs. control). Since IMCD cells express mostly calcium-dependent PKC, we use thapsigargin (1 μM), a non-competitive inhibitor of the sarco-endoplasmic reticulum Ca-ATPase (SERCA) to deplete cytoplasmatic Ca^2+^. As shown in Figure 5D, previous treatment with thapsigargin blocked the induction of PRR mediated by αKG (0.6 ± 0.1, *p* = 0.21 vs. control).

### *In silico* Studies Suggested Binding of αKG to Mouse OXGR1

**Figure 6:**
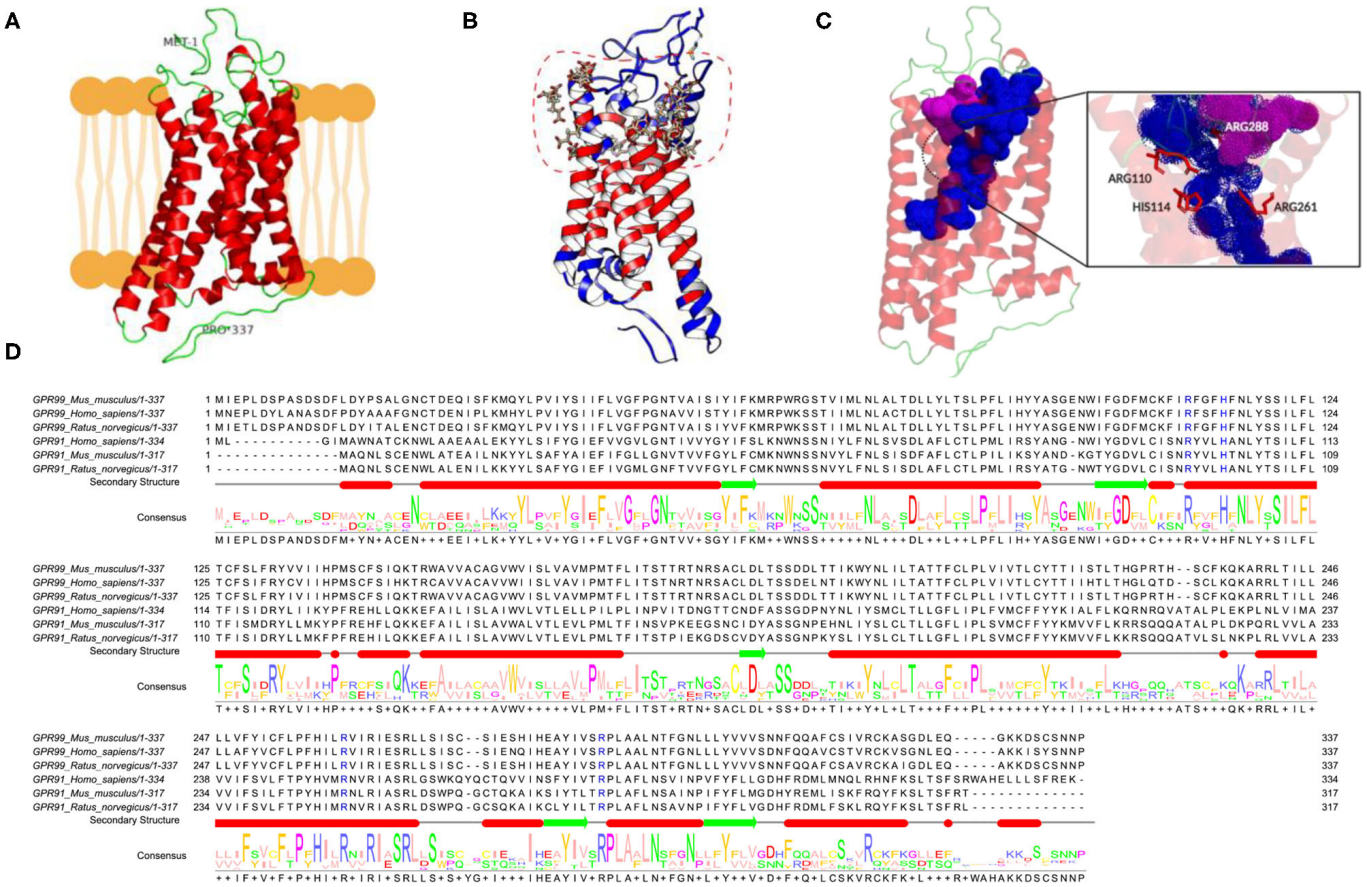
*In-silico* evidence of the binding of αKG to OXGR1 (GPR99). **(A)** Three-dimensional structure for mouse GPR99. **(B)** Using the server Swiss-Dock, the model in Panel **(A)** was used for docking studies of binding of αKG to OXGR1. **(C)** A large pocket inside the OXGR1 (blue color) can be observed in which ARG110, HIS114, ARG228, and ARG261 are the most important residues in the interaction with αKG. **(D)** Multiple alignment analysis in rat, mice, and human GPR99 (OXGR1) and GPR91 (P2Y) demonstrated that residues R99, H103, R252, and R281 of GPR91 are the same residues present in the hydrophobic pocket of OXGR1.

OXGR1 (GPR99) is a seven-transmembrane receptor. Due to the absence of a three-dimensional structure for mice, we used online server Phyre2, I-TASSER, and ProSA-Web and MolProbidity (Figure 6A). The model was used for docking studies of αKG to OXGR1 using the server Swiss-Dock (Figure 6B). As observed in the dotted line in Figure 6B, there are several binding sites located mainly at the extracellular side of the receptor. It should be mentioned that docking was performed using a rigid form of the receptor, which does not usually happen in nature. Figure 6C highlights the presence of a large pocket inside the OXGR1 (blue color) in which ARG110, HIS114, ARG228, and ARG261 are the most important residues interacting with αKG. OXGR1 shares a high identity with the P2Y receptor family, which can be activated by AMP and adenosine (36), as well as by αKG (22). By making a multiple alignment analysis with rat, mice, and human GPR99 (OXGR1) and GPR91 as shown in Figure 6D, the residues R99, H103, R252, and R281 of GPR91 are the same residues present in the hydrophobic pocket of OXGR1 (as seen in the magnification of the image in Figure 6C).

### OXGR1 Antagonism Suppressed HG-Dependent Induction of PRR in Inner Medullary Collecting Duct Cells

**Figure 7:**
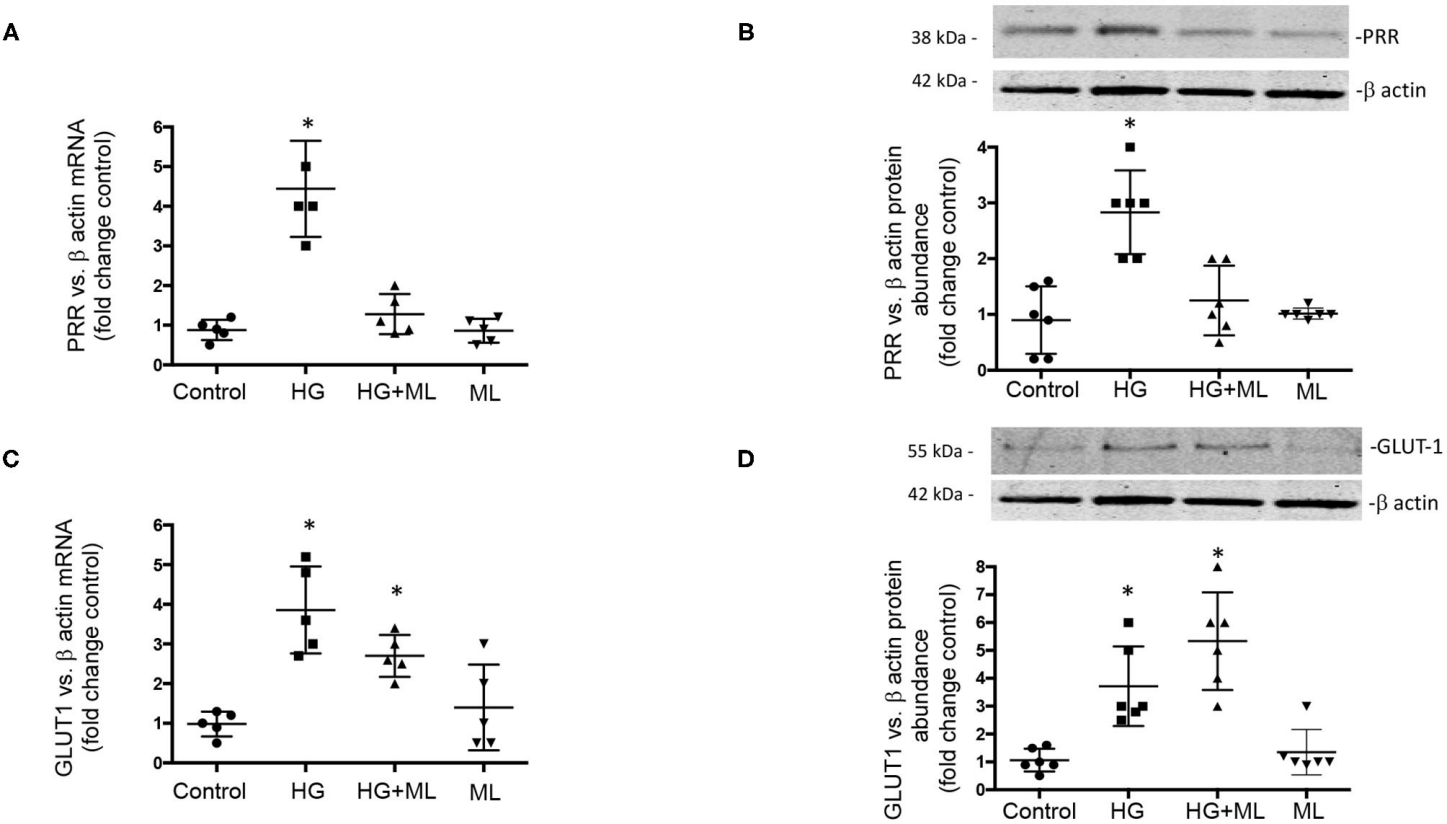
Effect of high glucose (HG) conditions (25 mM) on GLUT1 and (pro)renin receptor (PRR) expression in cultured inner medullary collecting duct (IMCD) cells subjected or not to OXGR1 pharmacological blockade. **(A)** PRR messenger RNA (mRNA) and **(B)** protein levels were augmented by 24-h HG treatment while pretreatment with 10^−7^ M of montelukast (ML) blunted the induction of PRR expression. Augmentation of GLUT1 at **(C)** mRNA and **(D)** protein levels was not altered by the pretreatment with ML. *p < 0.05 vs. control group, *n* = 5. Reblotting of blot **(B)** is shown in Panel **(D)**, and same representative β-actin loading control was used.

Finally, we evaluate the effect of HG conditions (25 mM) on GLUT1 and PRR expression in cultured IMCD cells subjected to OXGR1 pharmacological blockade. As shown in Figures 7A,B, PRR mRNA and protein levels were augmented by 24-h HG treatment (4.8 ± 0.9 vs. control 1.0 ± 0.1, *p* < 0.05), while pretreatment with 10^−7^ M of ML blunted the induction of PRR expression (1.2 ± 0.2, *p* = 0.08 vs. control). IMCD cells showed an increase in GLUT1 mRNA and protein (Figures 7C,D) levels under HG conditions (3.5 ± 0.5 vs. control 1.0 ± 0.2, *p* < 0.05). This phenomenon was not altered by the pretreatment with ML (3.6 ± 0.5, *p* < 0.05 vs. control). ML alone has no effect on GLUT1 or PRR expression as compared to controls.

## Discussion

We demonstrated that mice with 6 days of STZ-induced hyperglycemia exhibit augmentation of αKG in urine along with increased expression of PRR in medullary collecting ducts and augmented urinary AngII levels and Na^+^ retention. We also showed that pharmacological blockade of the αKG receptor, OXGR1, with ML *in vivo* and *in vitro* prevented the upregulation of PRR in medullary collecting duct cells observed during HG conditions. Moreover, blunted upregulation of PRR by ML was accompanied by attenuated increases in urinary excretion of AngII and increased natriuresis. These effects were independent of changes in blood pressure.

In the present study, we have used a model of early diabetic conditions to trigger with no gross kidney damage. Six days of hyperglycemia was chosen because longer periods implicate nephropathy and further damage (e.g., including collagen deposition in glomerular, tubulointerstitial, and perivascular areas) in rat (37) and mice kidneys after 3 and 5 weeks post-STZ injection, respectively (38). Furthermore, it is possible that ML treatment in STZ-induced diabetic mice for a longer period of time would result in further protection against PRR augmentation and PRR-related signaling pathways related to fibrosis; however, longer treatments (>6 days of single STZ dose) showed structural evidence of acute tubular necrosis in mice (39). We performed 24-h incubations with HG in primary cultures of IMCD cells because it has been reported that longer periods of incubation with HG significantly decreases the number of viable cells in cultured kidney cells (40). Additionally, our model of primary cultures of IMCD cells allows for experiments lasting up to 5 days. Beyond this time point, cells reach confluence and impair further treatment. Despite this, OXGR1 expression was not altered under HG conditions. However, prolonged exposure to high levels of αKG or HG *in vivo* may promote complex pathways that include receptor internalization and tissue damage.

PRR is a multifunctional protein (41) that is part of the multisubunit complex, vacuolar H^+^-ATPase, which plays a key role in intracellular acidification (42, 43), autophagy (44), and kidney development (45). PRR increases renin activity and fully activates prorenin (33, 46). Increased renin activity under pathological conditions has been further supported by *in vivo* data from different models of experimental hypertension demonstrating that PRR in the CD is required for the local formation of AngII (47, 48). Furthermore, Kang et al. demonstrated that CD is the main source of prorenin in STZ diabetic mice (2). More recent evidence has also shown that prorenin and renin levels are increased in the plasma and kidneys in diabetes (8, 11, 49). Our data corroborate the results of previous studies by demonstrating PRR augmentation in mesangial cells during HG conditions (50) and in CD during diabetes (51). HG was also shown to stimulate polarized translocation of PRR to the apical plasma membrane in proximal tubular HK-2 cells (52).

OXGR1 participates in paracrine communication between different parts of the renal tubules and is necessary for maintaining the systemic acid–base balance (53). We have shown that STZ treatment stimulates metabolic pathways that enhance αKG formation in CD. Since PRR is part of the vacuolar H^+^-ATPase, changes in its abundance may play an essential role in distal urine acidification and phenotype of intercalated cells, as described previously (54), which seems to be independent of changes in GLUT1 expression. Furthermore, ML treatment prevented increases in PRR expression in medulla and specifically intercalated cells but did not cause changes in GLUT1 expression, which may have otherwise altered intercalated cell function. Since αKG concentration in CD decreases in acute acidosis and increases in acute alkalosis, αKG/OXGR1 could be considered as a paracrine system allowing proximal and distal parts of the nephron to communicate. Furthermore, OXGR1 activation stimulate HCO_3−_ secretion and also contributes to NaCl reabsorption in earlier segments such as proximal tubules (55).

Because both PRR and renin are both increased in CD cells in diabetes, it is reasonable that both may contribute to distal AngII formation. Furthermore, levels of angiotensinogen, the substrate for renin, are also increased and secreted under diabetic conditions (56) contributing to AngI and AngII formation in the distal nephron. The concomitant augmented excretion of renin and increased expression of PRR leading to newly formed AngII supports the concept that intratubular RAS, which may have a differential expression between cortex and medulla in renal damage (57), confabulates to promote Na^+^ reabsorption impacting on blood pressure. Despite our evidence demonstrating that STZ mice exhibit Na^+^ retention, we were unable to demonstrate significant increases in arterial blood pressure on day 6 (Figure 1G). A slight but not significant increase was observed in systolic and diastolic blood pressure on day 6. This mild impact on blood pressure may be explained by an apparent negative body fluid balance, as suggested by the significant body weight loss on day 6 that was correlated with massive urine output in diabetic mice. Indeed, although body weight was reduced in STZ mice, weight gain evidenced by measuring body weight vs. tibia length may not explain the significant reduction in body mass. Furthermore, food intake was not altered by STZ treatment. Taken all together, it is possible that the increases in blood pressure may be clinically relevant after longer periods of diabetic disease, such that tubular damage is more evident as described previously (39). Despite this, it is clear that PRR upregulation in high glucose conditions may favor tubular RAS activation.

Despite the evidence of PRR upregulation in mesangial cells under HG conditions (13, 50, 58), the precise mechanisms involved in the regulation of PRR in the CD are not well-understood. Although tubular fluid under physiological conditions is virtually glucose free by the time it reaches the CD, basolateral uptake of glucose by GLUT1 facilitative transporters in the CD may stimulate the Kreb's cycle under diabetic conditions with consequent accumulation and secretion of Kreb's intermediaries (59). This idea is supported by studies showing that in STZ-diabetic rats, both the expression of GLUT1 in the CD and the levels of αKG in the urine are augmented (60).

In 2002, a new G-protein-coupled receptor with homology to a new subgroup of nucleotide receptors was described and called GPR99 (which is also known as 2-oxoglutarate receptor 1 or OXGR1) (22). Studies to deorphanize the OXGR1 revealed that αKG activates OXGR1 through Gq to increase intracellular Ca^2+^ (24). The EC50 of OXGR1 for αKG is in the millimolar range, similar to the concentration of αKG in the circulation (25), indicating that the ligand–receptor interaction is physiologically significant. Evidence has demonstrated that OXGR1 effects can be specifically inhibited by leukotriene receptor antagonists such as ML (23). In mice, OXGR1 is expressed only in the testes, smooth muscle cells, and predominantly in kidney CD cells (23). Double-labeling immunohistochemistry demonstrated the colocalization of OXGR1 in type B and non-A–non-B intercalated cells of the B1 subunit isoform of vacuolar H^+^-ATPase (25). PRR immunoreactivity is detected at the apical aspect of type A intercalated cells and non-A, non-B intercalated CD cells (61). Tokonami et al. demonstrated that millimolar concentrations of αKG act in a paracrine manner on type B and non-A–non-B intercalated cells stimulating NaCl reabsorption (25). These effects were induced by apical but not basolateral application of αKG and mediated by OXGR1. Based on this, it is likely that the increased levels of plasma and urine αKG described in diabetic animals can reach the CD at physiological concentrations high enough to activate OXGR1, stimulating signaling pathways to induce the expression of PRR. Furthermore, a recent revision from Peterdi et al. pointed out evidence of Kreb's cycle activation in the CD by HG (21) through enhanced GLUT1 expression (20), which in turn contributes to αKG secretion and augmented intratubular concentrations.

**Figure 8:**
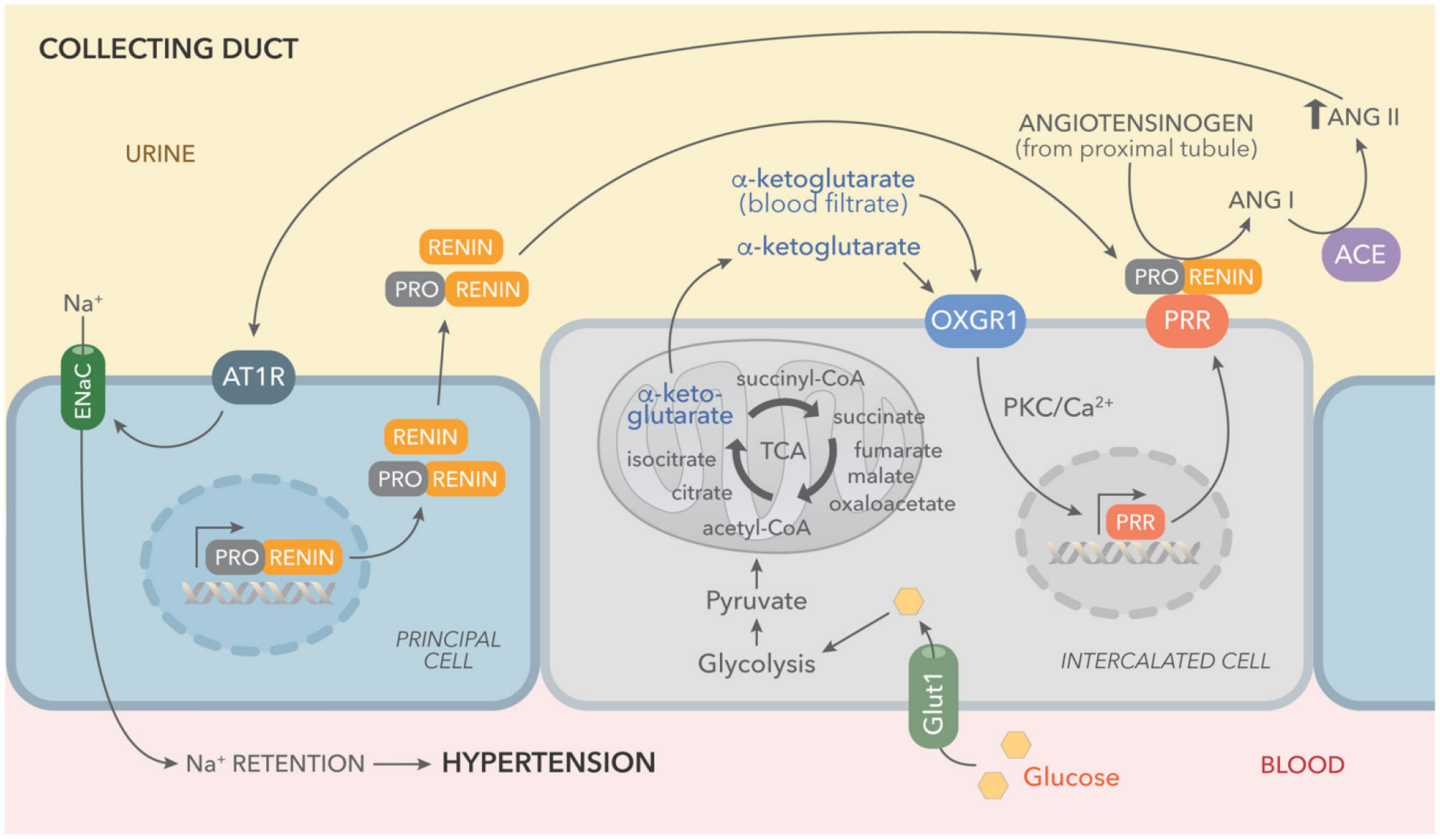
Working hypothesis representing the effects of high glucose on the activation of tricarboxylic acid (TCA) cycle and accumulation of αKG leading to OXGR1 activation. Elevated plasma glucose levels and increased expression of GLUT1 boost glycolysis and TCA cycle, causing αKG accumulation and secretion. Prorenin (and renin) released into the lumen by the principal cell, binds to (pro)renin receptor (PRR), consequently increasing renin activity and activating enzymatic activity of prorenin. Angiotensinogen in the urine (secreted by proximal tubules or coming from blood filtrate) is cleaved by activated prorenin or renin. Since ACE activity is present along the nephron, this intratubular RAS activation ends with AngII intratubular formation and angiotensin type 1 receptor (ATR1)-dependent stimulation of sodium reabsorption through epithelial sodium channels (ENaC).

This concept was supported by our results, which show increased levels of αKG in urine samples of STZ diabetic mice and in cultured IMCD cells (Figures 1, 5). We have reported that PRR is stimulated by the receptor 1 of AngII (AT1R), which is also a Gq-coupled receptor linked to protein kinase C activation and intracellular Ca^2+^ release (62). Higher levels of αKG might stimulate apical OXGR1, a Gq-coupled receptor that increases intracellular raising Ca^2+^ (Figure 5) and PKC activity (35). Thus, αKG coming from the filtrate and accumulation in collecting duct cells is able to stimulate OXGR1-dependent activation of PKC/Ca^2+^ pathways, which are responsible for the increased expression of PRR and further intratubular AngII formation (Figure 8).

This was further demonstrated in the experiments in which cultured IMCD cells were treated with physiological concentrations of αKG and consequently had increased intracellular Ca^2+^. We further confirmed these observations by blocking OXGR1 with ML. Preincubation with ML prevented the increase in intracellular Ca^2+^ levels in response to αKG treatment (Figure 5B). Furthermore, OXGR1 blockade prevented the increase in PRR mRNA and protein levels (Figure 5C). Interestingly, OXGR1 blockade did not affect GLUT1 expression in STZ mice or in cultured IMCD cells. Finally, we demonstrated that PKC inhibition and Ca^2+^ depletion impairs αKG-dependent augmentation of PRR (Figure 5D). Importantly, blockade of OXGR1 partially blunted the Na^+^ retention observed in STZ mice. This effect was observed during the first 3 h of the saline challenge test (Figure 3E). This was also accompanied by a partial reduction in urinary AngII formation observed in STZ mice (Figure 3).

Our data highlight the importance of the distal nephron segments in RAS activation and formation of intratubular AngII under conditions of HG. Our evidence also demonstrates that metabolic pathways might be involved in the regulation of intratubular RAS components.

## Data Availability Statement

The original contributions presented in the study are included in the article/Supplementary Material, further inquiries can be directed to the corresponding author/s.

## Ethics Statement

The animal study was reviewed and approved by Bioethical Committee of the Pontificia Universidad Católica de Valparaiso.

## Author Contributions

AG, BV, PC, SF, JV, PA, and NS-P performed experiments. QN and MK performed experiments and analyzed the data. CA and MP supervised the experiments. AG wrote the manuscript, supervised the experiments, and analyzed the data. CA, MP, QN, and MK approved the final form of the manuscript. All authors contributed to the article and approved the submitted version.

## Conflict of Interest

The authors declare that the research was conducted in the absence of any commercial or financial relationships that could be construed as a potential conflict of interest.
