# Supplementary material for: α-Ketoglutarate Upregulates Collecting Duct (Pro)renin Receptor Expression, Tubular Angiotensin II Formation, and Na+ Reabsorption During High Glucose Conditions
Source: Front Cardiovasc Med. 2021 Jun 4;8:644797. doi: 10.3389/fcvm.2021.644797 (PMC8220822; doi:10.3389/fcvm.2021.644797)

α-Ketoglutarate regulates collecting duct (pro)renin receptor, tubular angiotensin II formation and Na^+ reabsorption during high glucose conditions.

Aaron Guerrero, Bruna Visniauskas, Pilar Cárdenas, Stefanny M. Figueroa, Jorge Vivanco, Nicolas Salinas-Parra, Patricio Araos, Quynh My Nguyen, Modar Kassan, Cristián A. Amador, Minolfa C. Prieto, Alexis A. Gonzalez

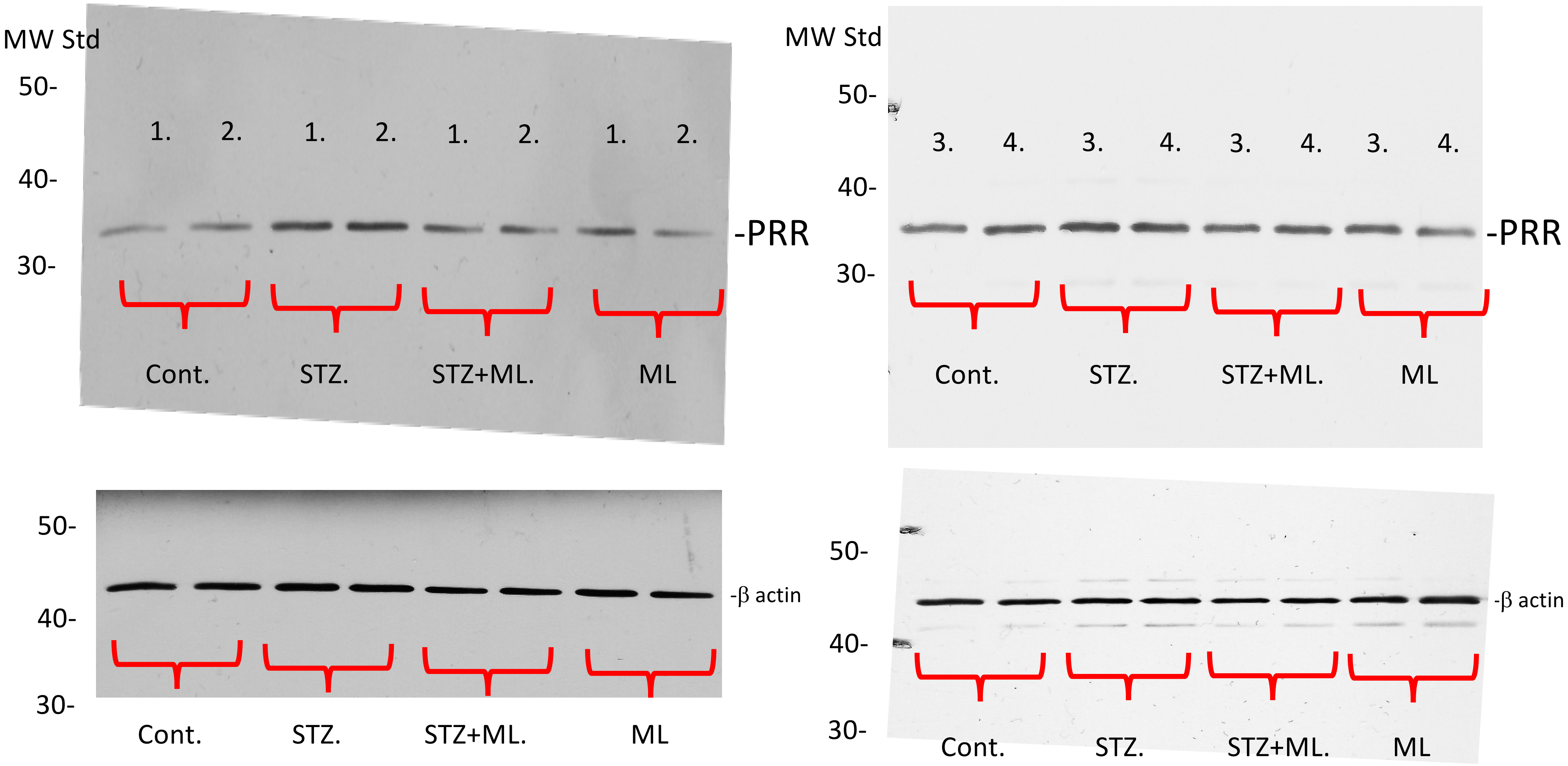

STZ mice tissue blots

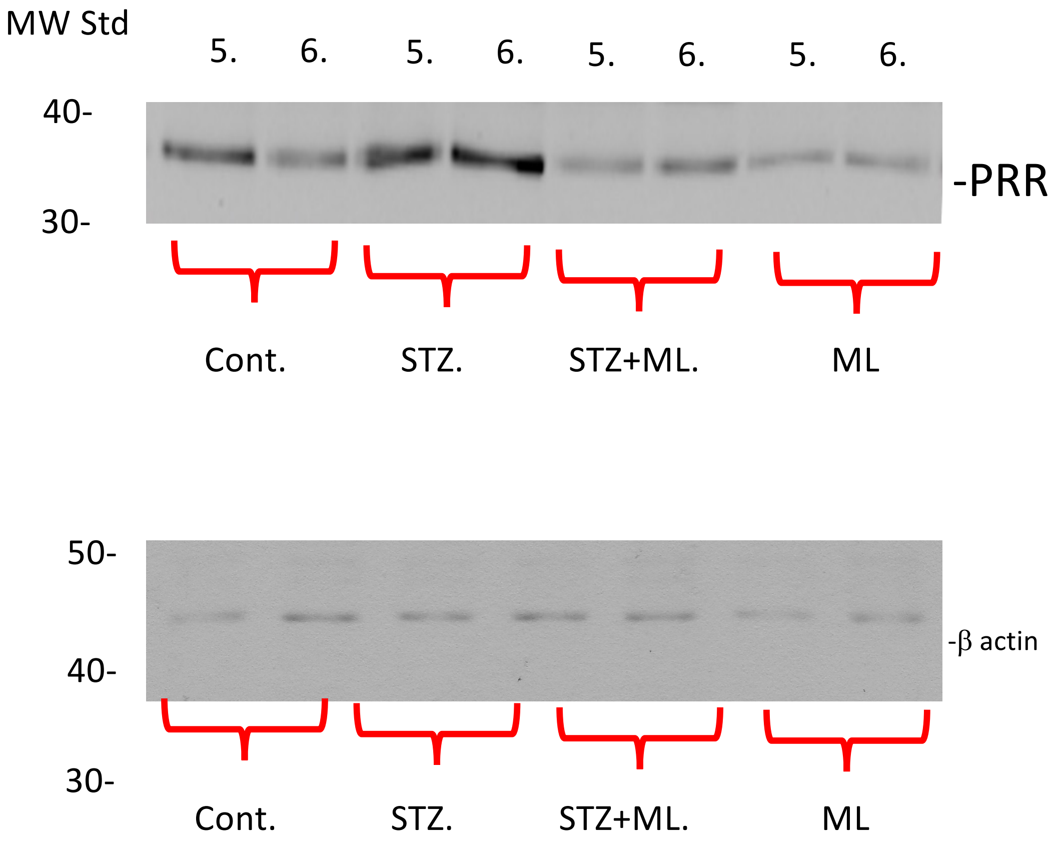

STZ mice tissue blots

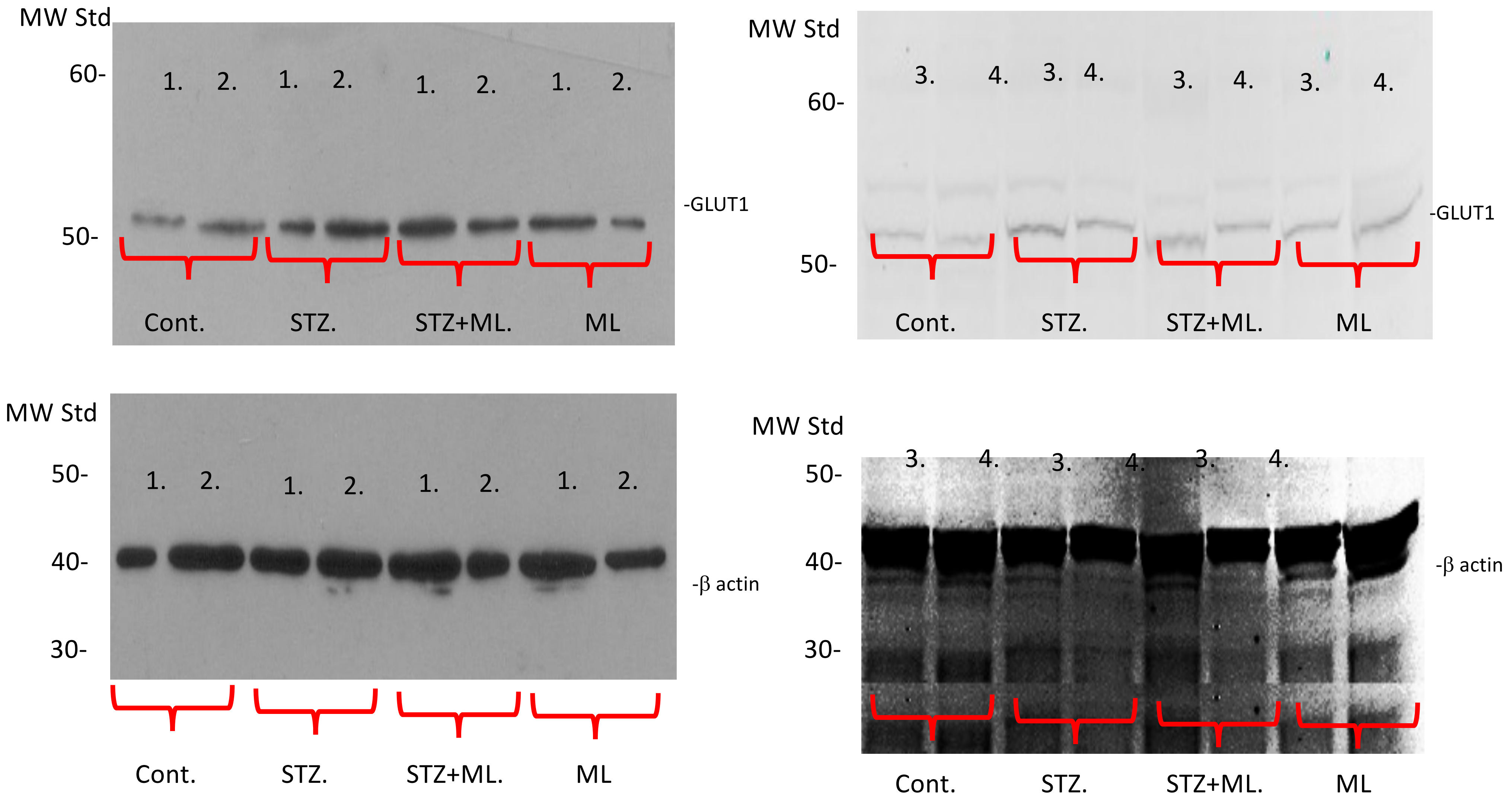

STZ mice tissue blots

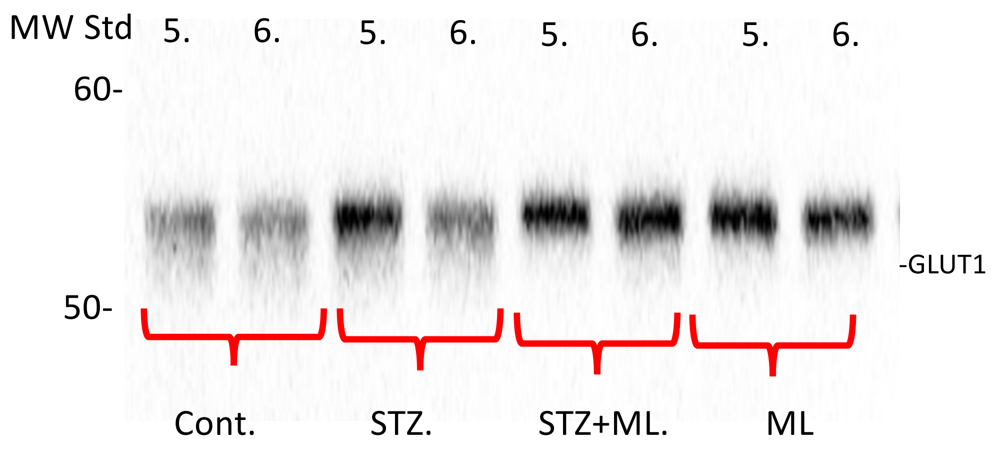

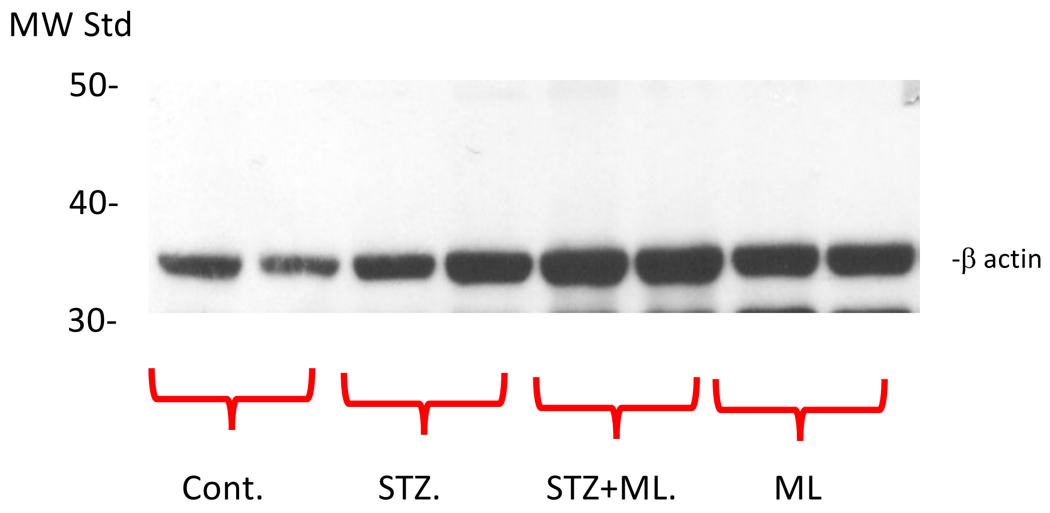

STZ mice tissue blots

HG in cultured IMCD cells

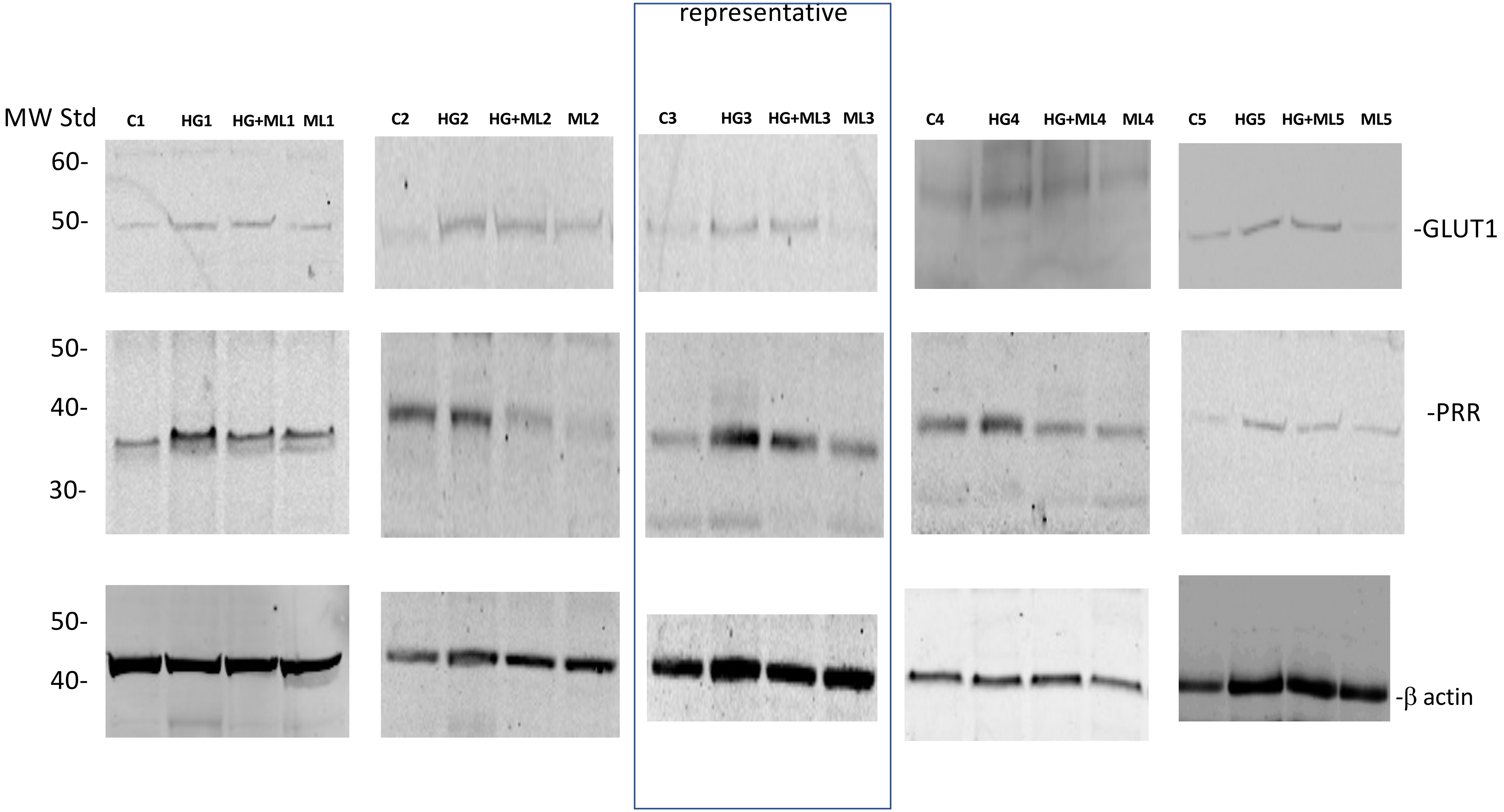

Alpha ketoglutarate in cultured IMCD cells

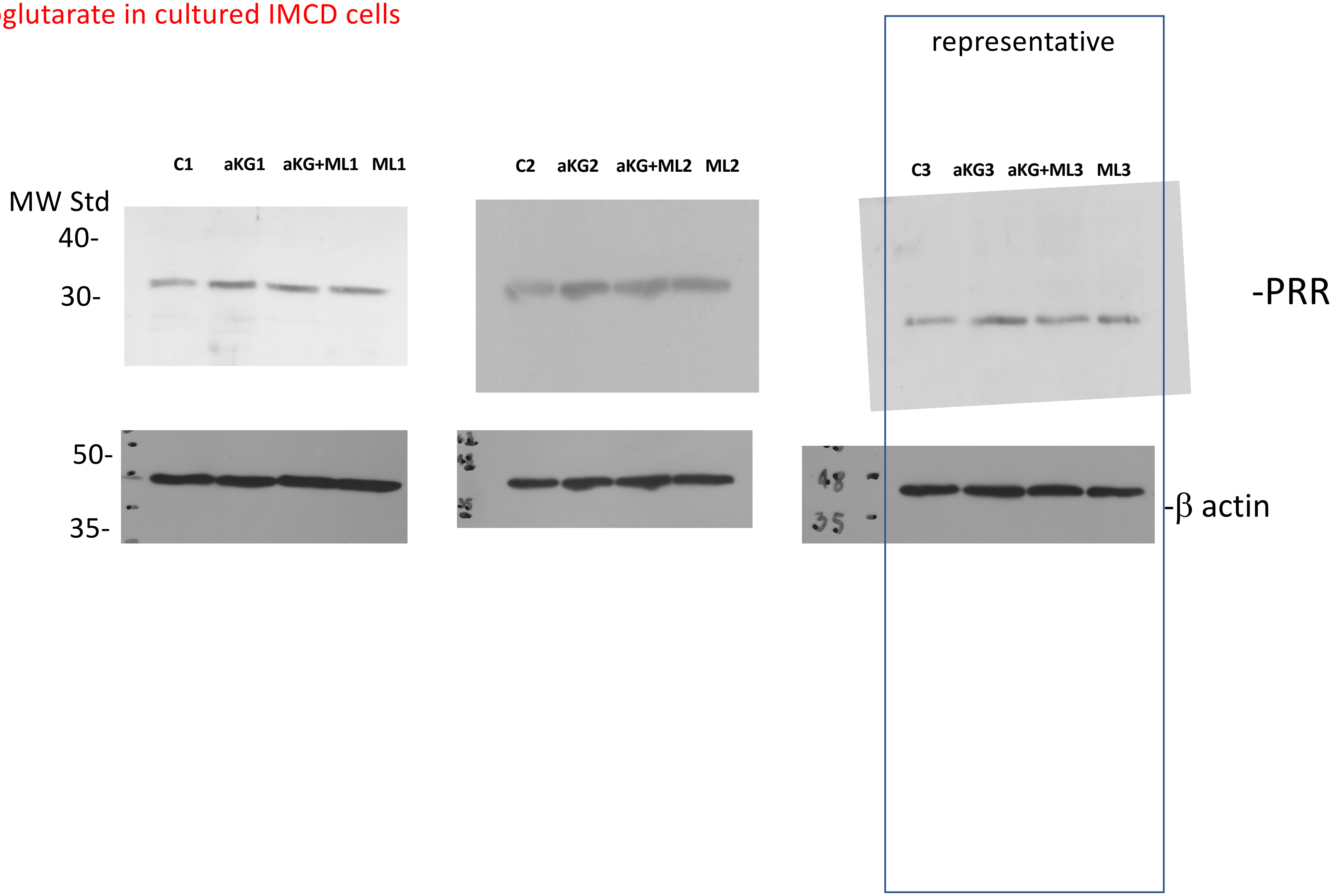

Alpha ketoglutarate in cultured IMCD cells and inhibition of PKC and blockade of intracellular Ca+

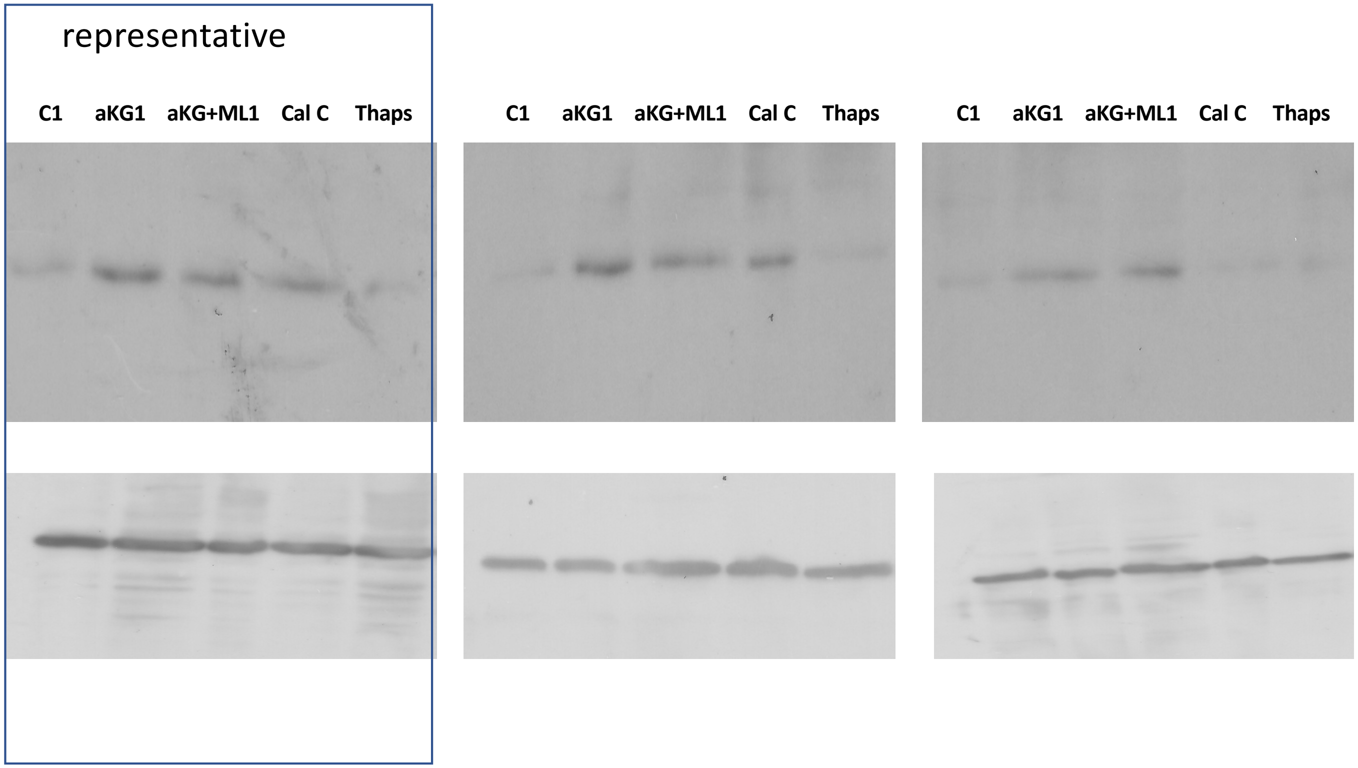

HG in cultured IMCD cells: effect on OXGR protein abundance

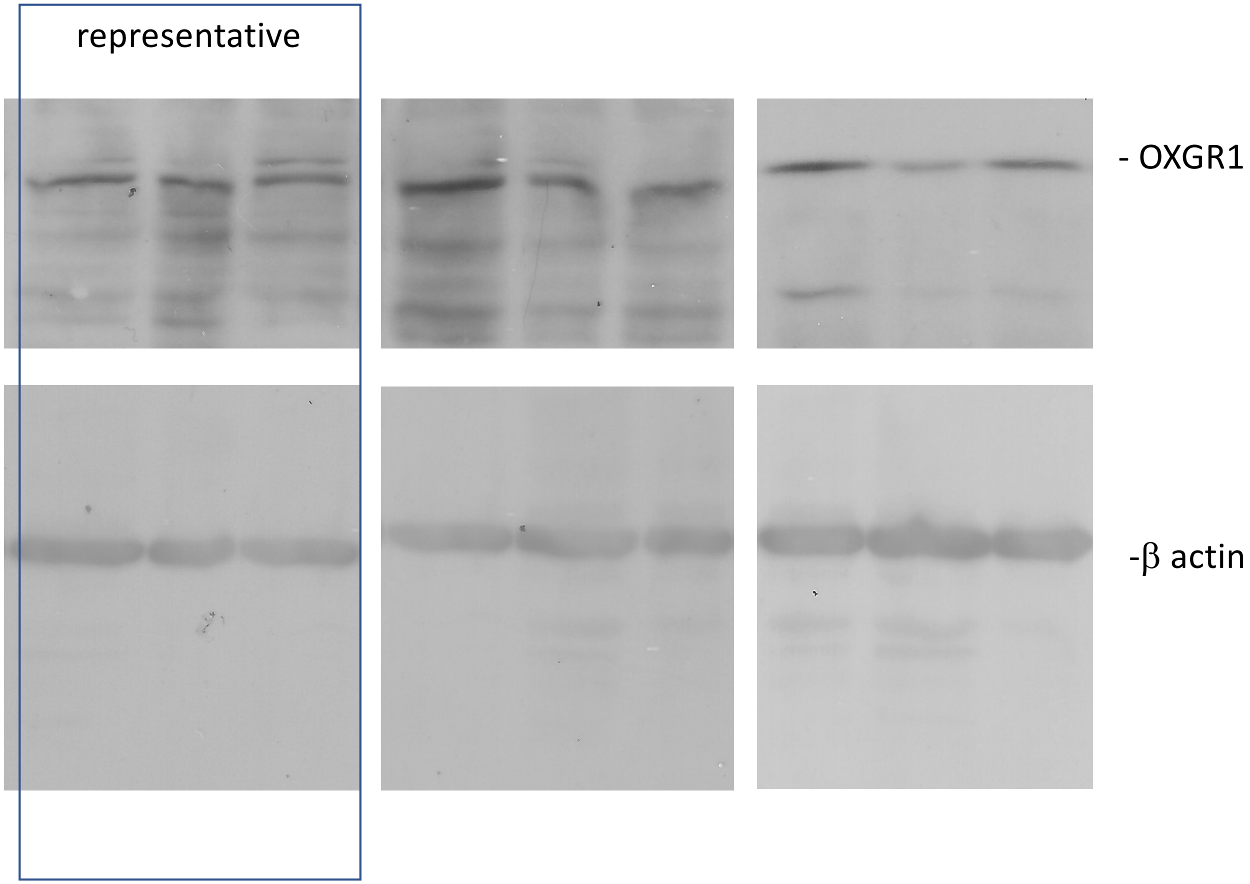

Supplement: Supplementary file 1 [file Data_Sheet_1.PDF]
